# Comparison of Clinical Characteristics and Outcomes of Hospitalized Patients Infected with the D614G Strain or Alpha Variant of COVID-19 in Taiwan: A Multi-Center Cohort Study

**DOI:** 10.7150/ijms.76725

**Published:** 2022-10-24

**Authors:** Allen Chung-Cheng Huang, Shu-Min Lin, Tzu-Hsuan Chiu, Ko-Wei Chang, Tse-Hung Huang, Tsung-Hsien Yang, Yi-Hsien Shiao, Chung-Shu Lee, Fu-Tsai Chung, Cheng-Hsun Chiu

**Affiliations:** 1 Department of Thoracic Medicine, Chang Gung Memorial Hospital, Chang Gung University, School of Medicine, Taoyuan, Taiwan.; 2 Department of Traditional Chinese Medicine, Chang Gung Memorial Hospital, Linkou, Taiwan.; 3 Department of Traditional Chinese Medicine, Chang Gung Memorial Hospital, Keelung, Taiwan.; 4 School of Traditional Chinese Medicine, Chang Gung University, Taoyuan, Taiwan.; 5 Graduate Institute of Health Industry Technology, Research Center for Chinese Herbal Medicine, Chang Gung University of Science and Technology, Taoyuan, Taiwan.; 6 Department & Graduate Institute of Chemical Engineering & Graduate Institute of Biochemical Engineering, Ming Chi University of Technology, New Taipei, Taiwan.; 7 School of Nursing, National Taipei University of Nursing and Health Sciences, Taipei, Taiwan.; 8 Department of Traditional Chinese Medicine, New Taipei Municipal Tucheng Hospital, New Taipei City, Taiwan.; 9 Department of Pulmonary and Critical Care Medicine, New Taipei Municipal Tucheng Hospital, New Taipei City, Taiwan.; 10 Division of Pediatric Infectious Diseases, Department of Pediatrics, Chang Gung Memorial Hospital, Chang Gung University College of Medicine, Taoyuan, Taiwan.

**Keywords:** COVID-19, D614G, Alpha variant

## Abstract

**Objective:** Direct comparison of the clinical traits of coronavirus disease 2019 (COVID-19) in strain D614G, which originated from Wuhan, China, and the Alpha variant, which contains 17 mutations, infected patients could help physicians distinguish between strains and make clinical decisions accordingly. This study sought to compare the clinical characteristics and outcomes of the D614G strain and Alpha variant of SARS-COV-2 and identify the predictors for viral RNA clearance and in-hospital mortality in patients with COVID-19.

**Methods:** This study recruited consecutive patients from four hospitals between March 1, 2020, and July 31, 2021. Demographic characteristics, laboratory results, and clinical outcomes were determined.

**Results:** Among the 239 enrolled patients, 11.2% (27/239) were infected with strain D614G and 88.7% (212/239) were infected with the Alpha variant. There were no significant differences in disease progression, rate of respiratory failure, subsequent development of acute respiratory distress syndrome (ARDS), acute kidney injury, cardiac injury, duration of stay in the intensive care unit or hospital, discharge rate, mortality rate, or viral RNA clearance time between the two groups. Multivariate Cox regression revealed that antibiotic therapy reduced the risk of delayed viral RNA clearance (hazard ratio [HR], 0.26; 95% confidence interval [CI], 0.13-0.55), while autoimmune disease increased the risk of delayed viral RNA clearance (HR, 3.98; 95% CI, 1.21-13.04). Elderly patients (age > 65 years) and patients with a history of cerebrovascular accident (CVA) were at increased risk of in-hospital mortality (HR, 5.14; 95% CI, 1.06-24.72 and HR, 3.62; 95% CI, 1.25-10.42, respectively).

**Conclusions:** There were no significant differences between the D614G strain and Alpha variant of COVID-19 in terms of clinical characteristics and outcomes. However, factors affecting viral RNA clearance and the risk of in-hospital mortality were identified. These results could help to inform the future prioritization of resource allocation and identify patients in need of intense monitoring.

## Introduction

The emergence of coronavirus disease 2019 (COVID-19) has had an extensive impact on public health and economies globally. As of April 2022, there have been more than 494 million confirmed COVID-19 cases worldwide, with more than 6.1 million deaths attributed to the pandemic (https://covid19.who.int./). Taiwan was one of the few countries to achieve initial success in COVID-19 control without strict city lockdowns or school closures. Based on past experience of combating the severe acute respiratory syndrome coronavirus-1 (SARS-COV-1) outbreak in 2003, Taiwan responded to the COVID-19 pandemic with rapid measures as early as January 2020, resulting in only a few scattered household and workplace transmissions 1. Most confirmed cases in Taiwan were imported from other countries, while further genetic sequencing confirmed that the D614G strain originated from Wuhan, China. The D614G strain, which emerged as the dominant strain globally in 2020, was associated with increased transmissibility but without increased incidence of severe illness 2. However, when a country-wide outbreak started in Taiwan in May 2021, the Alpha variant was identified as the dominant strain of transmission rather than the D614G strain.

SARS-COV-2 is prone to genetic evolution, resulting in multiple variants that may exhibit different characteristics compared to ancestral strains. In late 2020, a variant of concern (VOC) known as lineage B.1.1.7, also referred to as the Alpha variant or GRY (GY/501Y.V1), was first reported based on whole-genome sequencing of samples from patients testing positive for SARS-COV-2 3, 4. The Alpha variant of SARS-CoV-2 exhibits 17 mutations, of which eight are found in the spike protein that mediates viral attachment and entry into human cells. At least three mutations potentially affect viral function. Mutation N501Y is a key contact residue in the receptor binding domain and enhances viral binding affinity to angiotensin-converting enzyme 2 (ACE2) 5, 6. This VOC had been circulating in the United Kingdom (UK) since September 2020 and was reported to be 43% to 82% more transmissible, surpassing preexisting strains of SARS-COV-2 and emerging as the dominant strain 7. The aforementioned outbreak of the COVID-19 Alpha variant in Taiwan in 2021 resulted in more than 500 deaths before coming to an end in August 2021. While strain D614G and the Alpha variant were each dominant in different parts of the world at one point, direct comparison of the two variants in terms of clinical characteristics and outcomes appeared somewhat lacking. Identifying differences in clinical traits in strains could help physicians to make clinical decisions based on the particular infecting strain. Therefore, we conducted a study to compare and contrast clinical characteristics and outcomes in COVID-19 patients infected with the D614G strain or the Alpha variant and also sought to identify the factors associated with SARS-COV-2 viral RNA clearance and in-hospital mortality.

## Materials and methods

### Study Population

This study reports on a multi-center, retrospective, observational study of subjects hospitalized due to COVID-19 and managed within the Chang Gung Health System. We recruited consecutive patients from four hospital branches, including two large tertiary care centers and two reginal hospitals. Subjects were included if they were > 18 years old and had had a laboratory-confirmed diagnosis of COVID-19 via polymerase chain reaction (PCR) testing. Data were collected for 239 consecutive patients admitted to the Chang Gung Health System between March 1, 2020, and July 31, 2021. In addition, 89 hospitalized patients during the same study period with the diagnosis of non-COVID-19 pneumonia were also collected for comparison. Due to the outbreak of COVID-19 pandemic, most of the medical wards were reassigned as quarantine wards. The hospitals only accepted referred non-COVID-19 pneumonia patients with at least one organ failure. Those non-COVID-19 pneumonia patients without organ failure were admitted to community hospitals. The study non-COVID pneumonia was approved by the Institutional Review Board of the Chang Gung Memorial Foundation (IRB No. 202100712B0). Informed consent was waived due to the retrospective nature of this study. All data were collected from electronic medical records.

### Data Collection

Medical information, including baseline characteristics and comorbidities, as well as clinical, laboratory, treatment, and outcome data, was extracted using data collection forms, which were then checked independently by two trained physicians.

### Definitions

All of the patients included in the study were diagnosed with COVID-19 in accordance with the guideline released by the Taiwan Centers for Disease Control (CDC): “Interim Guidelines for Clinical Management of SARS-CoV-2 Infection” 8. There have been two waves of COVID-19 pandemic in Taiwan. The first wave occurred from January 2020 to January 2021, and the second wave occurred from May 2021 to July 2022. According to the report from whole genome sequencing by Taiwan CDC, the first wave of COVID-19 patients were infected with strain D614G while the second wave of COVID-19 patients were infected with the Alpha variant.

The patients were accordingly classify as (1) mild cases, involving mild clinical symptoms without manifestation of pneumonia in imaging; (2) moderate cases, involving fever and respiratory tract symptoms as well as manifestation of pneumonia in imaging; (3) severe cases, involving any of the following: (i) respiratory distress with respiratory rates ≥ 30 breaths/minute, (ii) oxygen saturation of ≤ 94% in resting state, (iii) an arterial oxygen tension (PaO_2_) over inspiratory oxygen fraction (FiO_2_) ratio of < 300 mmHg (1 mmHg = 0.133 kPa), (iv) multiple pulmonary lobes with images showing progression in more than 50% of lesions within 24-48 h; and (4) critically ill cases involving (i) respiratory failure requiring mechanical ventilation, (ii) shock, and (iii) multiple organ failure requiring monitoring and treatment in the intensive care unit (ICU). Progression was defined as an increase in severity, while unchanged severity throughout the observation period was not classified as progression. Acute kidney injury (AKI) events were defined as any of the following criteria occurring within 7 days after admission: an increase in serum creatinine level of **≥** 0.3 mg/ dL within a period of 48 h or an increase in serum creatinine level of **≥** 1.5 times from baseline within 7 days. Note that both of these criteria were suggested in “Kidney Disease: Improving Global Outcomes (KDIGO) Clinical Practice Guideline for Acute Kidney Injury” 9. Cardiac injury was defined by at least one documented assay indicating elevated high sensitivity troponin-I 10.

### COVID-19 Management Protocol

The strategies used in the management of COVID-19 were fairly homogenous across the four hospitals, all of which adopted protocols in line with the “Interim Guidelines for Clinical Management of SARS-CoV-2 Infection”. This involved regularly monitoring vital signs and oxygen saturation (severe cases were monitored continuously), strengthening supportive treatment, providing sufficient calories, and maintaining the stability of the internal environment (e.g., water, electrolytes, and acid-base balance). Supplemental oxygen therapy was immediately administered to patients upon hypoxemia presentation. The target oxygen saturation was a pulse oxygen saturation of ≥ 90%. In the event that standard oxygen therapy failed, high-flow nasal catheter oxygen or non-invasive ventilation was used, while invasive mechanical ventilation was initiated if non-invasive mechanical ventilation failed to provide benefits. Anti-viral therapy and anti-inflammatory agents were administered in accordance with standardized guidelines. Remdesivir was administered to patients with SPO_2_ ≤ 94 % under room air or supplied oxygen. Low-dose dexamethasone (6 mg per day for no more than 10 days) was administered to patients with SPO_2_ ≤ 94 % under room air or supplied oxygen, patients presenting respiratory failure, and patients requiring extracorporeal membrane oxygenation (ECMO). Tocilizumab was administered to patients with SPO_2_ < 94 % under room air or supplied oxygen, as well as those presenting with respiratory failure or requiring ECMO. Antimicrobial agents (oral or intravenous) were prescribed in accordance with the condition of the patient. Anti-viral and anti-inflammatory agents were provided by the Taiwan CDC in accordance with standard guidelines. It should be noted that all medical expenses are reimbursed by national health insurance in Taiwan.

### Laboratory Data

All blood samples were obtained and analyzed using standardized laboratory methods. Routine hematological and biochemical testing included white blood cell count (WBC), lymphocyte count, prothrombin time (PT), activated partial prothrombin time (aPTT), C‐reactive protein (CRP), D-dimer, lactate dehydrogenase (LDH), ferritin, interleukin-6 (IL-6), and liver and kidney function tests. Repeat PCR testing was conducted at intervals decided by individual managing physicians. The PCR cycle threshold (Ct) values of nasopharyngeal samples were measured.

### Statistical Analysis

All data were expressed as mean ± standard deviation or percentages unless otherwise indicated. The Student's t-test was used to compare the means of continuous variables and normally distributed data; otherwise, the Mann-Whitney test was used. Categorical variables were tested using the Chi-square test or Fisher's exact test. We implemented the Kaplan-Meier curve and log-rank test to compare in-hospital mortality and viral RNA clearance; the latter was defined as the time between the onset of symptoms to two consecutive PCR Cts > 30 since Ct values above this cut-off have been associated with a lack of viral culture 11, 12. Univariate analysis was first performed to identify the predictors for viral RNA clearance and in-hospital mortality. All variables with a p-value of **<** 0.1 in univariate Cox regression analysis were entered into a multivariate Cox regression model to identify factors independently predictive of viral RNA clearance and in-hospital mortality A p-value of **<** 0.05 was considered statistically significant. All statistical analysis was performed using SPSS software, version 24.0 (SPSS, Inc., Chicago, IL).

## Results

### Demographic Data

During the study period, 239 patients with COVID-19 met the inclusion criteria. Among them, 27 patients (11.2%) were infected with strain D614G and 212 patients (88.7%) were infected with the Alpha variant. Out of all the patients, 100 (41.8%) had severe disease, and 40 (16.7%) had critical disease. The mean age of the patients was 59.1 ± 14.5 years, and approximately half of them were male (50.6%). Eleven patients (4.6%) were active smokers. Hypertension was the most common comorbidity (37.2%) among all studied patients with COVID-19. Sixteen patients (6.7%) received high-flow nasal cannula therapy during the hospitalization period. The majority of the patients (70.7%) underwent antibiotic therapy, but only patients infected with the Alpha variant received anti-IL-6 therapy (31.1%). A dose of 6 mg dexamethasone per day was administered to patients who required any form of oxygenation therapy (165 patients; 69%). Patients infected with the Alpha variant presented with significantly lower lymphocyte counts (1058.2 ± 666.1 vs 1613.4 ± 1049.5, p = 0.014) and higher LDH values (371.1 ± 189.8 vs 270.7 ± 142.5, p = 0.041) than patients infected with D614G strain, as shown in Table 2. Detailed demographic and clinical characteristics of all included patients are presented in Tables 1 and 2.

### Clinical Outcomes

**Figure 1:**
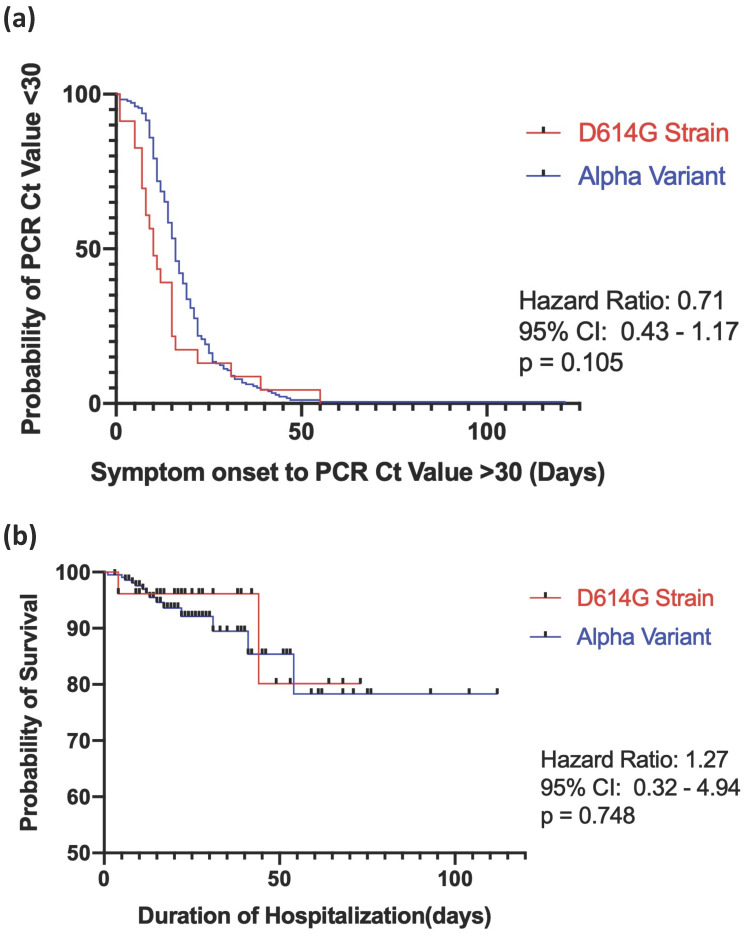
Kaplan-Meier survival curve for D614G vs the alpha variant in **(a)** SARS-COV-2 RNA clearance and **(b)** in-hospital mortality. Viral RNA clearance is defined as the time between the onset of symptoms to two consecutive PCR Cycle threshold>30. Log rank test p<0.05 indicates a statistical significance.

The results revealed that 94 hospitalized patients with COVID-19 (39.3%) experienced progression of the disease. A total of 65 patients (27.2%) experienced respiratory failure, and 44 (18.4%) subsequently developed ARDS. The mean durations of ICU and hospital stays were 23.0 ± 22.0 days and 23.0 ± 32.0 days, respectively. The in-hospital mortality rate was 7.1%. There were no significant differences between the D614G strain and Alpha variant groups with respect to respiratory failure rate or subsequent ARDS, AKI, cardiac injury, discharge rate, mortality rate, or duration of time spent in the ICU or hospitalized, as shown in Table 3. The Kaplan-Meier survival curve comparing viral RNA clearance for strain D614G and the Alpha variant is illustrated in Figure 1a, and in-hospital mortality is shown in Figure 1b. There were no statistical differences between the two groups in regard to viral RNA clearance time (hazard ratio [HR], 0.71, ref. Alpha variant; 95% confidence interval [CI], 0.43-1.17, log-rank test p = 0.105) or in-hospital mortality (HR, 1.27, ref. Alpha variant; 95% CI, 0.32-4.94, log-rank test p = 0.748).

### COVID-19 vs Non-COVID-19 Patients

Our study was conducted in tertiary referral centers and reginal hospitals. Due to the COVID-19 pandemic, we only admitted non-COVID-19 pneumonia patients with at least one organ failure. The hospital capacity for the non-COVID-19 patients was markedly reduced, and medical resources were re-allocated towards quarantine wards. The non-COVID-19 patients were predominantly male (69.7% vs 50.6%), with significantly higher rate of malignancy and immunosuppressive therapy (48.3% vs 3.8% and 36.0% vs 1.3%, respectively). Compared with COVID-19 patients, increased rate of respiratory failure (73.0% vs 27.2%), longer length of hospital stay (33.8 ± 31.1 vs 23 ± 32 days), and subsequently higher mortality rate (55.1% vs 7.1%) were observed in the non-COVID-19 group (Table 4).

### Univariate and Multivariate Cox Regression

Univariate analysis (Table 5) shows that for strain D614G, age > 65, CVA, autoimmune disease, dexamethasone, and antibiotics therapy were primarily selected. Furthermore, multivariate Cox regression analysis identified autoimmune disease history (adjusted HR, 3.98; 95% CI, 1.21-13.03, p = 0.022) as a positive predictor, and antibiotic therapy (adjusted HR, 0.26; 0.13-0.55, p < 0.001) as a negative predictor for delayed viral RNA clearance.

After performing univariate and multivariate Cox regression analysis, age > 65 years (adjusted HR, 5.14; 95% CI, 1.06-24.72; p = 0.041) and history of CVA (adjusted HR, 3.62; 95% CI, 1.25-10.42; p = 0.017) were the only two independent factors associated with increased in-hospital mortality in patients with COVID-19 (Table 6).

## Discussion

The results of this study showed that for patients infected with strain D614G or the Alpha variant of COVID-19, viral RNA clearance times, discharge rates, mortality rates, and duration of ICU or hospital stays did not differ significantly. This is in contrast to other clinical studies; for example, a large matched cohort study reported that mortality hazard was 1.64 times higher in patients infected with the Alpha variant than in patients infected with the previously circulating strains in the UK 13. Furthermore, two other studies reported HRs of 1.61 and 1.67 versus non-Alpha strains 14, 15. Although these previous studies demonstrated a higher risk of mortality with the Alpha variant, the administration of proven clinical therapies such as anti-IL-6, remdesivir, and dexamethasone at the time of the Alpha outbreak in Taiwan in mid-2021 may have played a role in reducing the mortality rate. Our results also indicated that patients infected with the Alpha variant presented with significantly lower lymphocyte counts and higher LDH levels than patients infected with strain D614G. SARS-CoV-2 infection often results in multiple organ injury accompanied by elevated levels of serum inflammatory mediators, indicating that COVID-19 is a systemic inflammatory illness rather than simply a lung disease 16. Another study reported that elevated LDH levels were associated with a six-fold increase in the odds of developing severe disease for patients with COVID-19 17. Since lymphocyte count and LDH levels are both markers of inflammation and correlate with the severity of the disease, the higher incidences of severely and critically ill individuals found in our Alpha variant group, compared with the D614G group (43.4% vs. 29.6% vs and 17.5% vs. 11.7%, respectively, see Table 1) may have contributed to our finding.

In order to effectively allocate resources and adjust quarantine policy accordingly, it is important to identify prognostic factors for SARS-COV-2 RNA clearance. In our current study, we found that the presence of autoimmune disease in COVID-19 patients was associated with a four-fold increase in the risk of delayed viral RNA clearance. Past studies have shown that inflammatory bowel disease and rheumatological diseases are associated not only with an increased risk of community-acquired and opportunistic infections but also with higher mortality, especially for patients undergoing some form of immunosuppressive therapy 18, 19. Several reports in the earlier phase of the pandemic also demonstrated that immunocompromised individuals are prone to have longer period of shedding of SARS-CoV-2. During the prolonged viral RNA shedding, some within-host viral evolution was observed. Potential factors contributing to the delayed viral clearance are the compromised immune status of the host and viral genetic evolution 20, 21. Therefore, this evidence may support our finding that patients with autoimmune diseases under immunosuppressants were predisposed to prolonged periods of SARS-CoV-2 viral clearance.

Although the routine use of antibiotics has been controversial in treating COVID-19 patients, use of adequate broad-spectrum antibiotic therapy helps to prevent and manage secondary bacterial infections and sepsis 22-24. We found patients receiving antibiotics to have a decreased risk of delayed viral clearance. While it is difficult to examine the exact microbial and immunological response, as well as the clinical benefits of antibiotics, past studies have shown that early administration of macrolides (such as azithromycin), which possess immune-modulating properties, helps to prevent severe lower respiratory tract illnesses in viral infections 25. A study by Du *et al*. reported azithromycin demonstrated *in vitro* anti-viral effect against SARS-COV-2 and blocks the entry of SARS-CoV-2 in HEK293T-ACE2 and Caco2 cells 26. Another observational study also reported the clinical benefits of antibiotics: the combination of remdesivir-azithromycin treatment significantly decreased the ICU admission rate 27. However, the exact mechanism of antibiotics in SARS-COV-2 viral clearance remains to be investigated.

The infectivity of COVID-19 patients is determined by the presence of virus in bodily fluids, secretions, and excreta 28; therefore, all patients with positive viral RNA detection need to be isolated to prevent further transmission. However, this policy has a huge impact in terms of medical and economic resources. Previous studies showed that successful viral culture was associated with PCR Ct values < 30; therefore, a PCR Ct value > 30 may be considered as a surrogate indicator for the end of the infective period. In many countries, isolated COVID-19 patients can be de-isolated after the relief of symptoms and two successive RNA Ct values > 30 in respiratory specimens 8, 29. Our study demonstrated that patients presenting autoimmune disease as a comorbidity and lacking treatment with antibiotics are strong candidates for delayed viral clearance and should therefore be given priority in the allocation of resources. The incorporation of these variables into clinical and laboratory algorithms could potentially be useful for future research.

We also found that elderly patients aged > 65 years and patients with a history of stroke were at increased risk of in-hospital mortality. A recent meta-analysis of 14 studies identified age > 65 years to be one of the risk factors associated with mortality in COVID-19, with a pooled odds ratio of 4.59 30. Age-related defects in B-cells and T-cells in elderly patients could result in prolonged inflammatory responses and deficient viral clearance, leading to eventual death 31. In addition, elderly patients with COVID-19 may have more pre-existing comorbidities and risk factors, including cerebrovascular disease 32, 33. The results of the current study could help clinicians to identify and be mindful of high-risk patients who require more intensive monitoring and intervention. Although our results demonstrated no difference in clinical outcomes in hospitalized patients in D614G and the Alpha variant, taking into consideration that the treatment and preventive measures for COVID-19 evolved over time, the identified risk factors for disease progression and in-hospital mortality may still remain and be applicable to the current Omicron pandemic and future variants of concern.

One major limitation of the current study is its retrospective nature, which may have led to bias in the selection of study subjects. Moreover, the sample size was rather small, especially for patients infected with D614G; therefore, the results should be interpreted with caution. Nonetheless, it should be noted that in Taiwan, all confirmed cases before the Omicron pandemic were admitted to hospital for treatment, based on guidelines declared by the Taiwan CDC. This made it possible for us to follow up patients longitudinally and maintain consistency among hospitals in terms of treatment regimens. Furthermore, the non-COVID-19 patients during the same study period were more critically ill than COVID-19 patients. The admission criteria of non-COVID-19 pneumonia patients with at least one organ failure may lead to some selection bias. However, our results demonstrated the “crowding-out effect” on other diseases that require in-patient treatment when a major pandemic outbreak occurs in the real-world setting.

In conclusion, there were no significant differences in clinical characteristics and outcomes for the D614G strain and Alpha variant of COVID-19. However, we successfully identified that antibiotic therapy could potentially reduce the risk of delayed SARS-COV-2 RNA clearance, while patients with autoimmune disease were shown to be at increased risk of delayed viral RNA clearance. Furthermore, elderly patients and patients with a history of CVA were at increased risk of in-hospital mortality. These findings could inform the future prioritization of resource allocation and identify patients in need of intense monitoring.

## Figures and Tables

**Table 1:** Baseline characteristics of COVID-19 patients

	Total n = 239	D614G Strain n = 27	Alpha Variant n = 212	p value
Age, yr	59.1 ±14.5	53.7 ± 19.5	59.8 ± 17.2	0.088
Male gender	121(50.6)	9(33.3)	112(52.8)	0.505
BMI	25 ± 4.7	26.1 ± 5.5	24.8 ± 4.6	0.303
Active smoker	11(4.6)	0(0)	11(5.2)	0.001
**Comorbidities**				
Hypertension, n(%)	89(37.2)	8(29.6)	81(38.2)	0.086
CAD	35(14.6)	1(3.7)	34(16.0)	0.008
Heart failure	10(4.2)	1(3.7)	9(4.2)	0.895
Atrial fibrillation	10(4.2)	0(0)	10(4.7)	0.001
CVA	12(5.0)	3(11.1)	9(4.2)	0.286
DM	48(20.1)	1(3.7)	45(21.2)	0.144
CKD	21(8.8)	3(11.1)	20(9.4)	0.181
COPD	11(4.6)	0(0)	11(5.2)	0.001
Asthma	7(2.9)	1(3.7)	6(2.8)	0.801
Autoimmune disease	3(1.3)	0(0)	3(1.4)	0.536
Malignancy	9(3.8)	1(3.7)	8(3.8)	0.986
HIV (+)	6(2.5)	0(0)	6(2.8)	0.990
**Therapies**				
High flow nasal cannula	16(6.7)	1(3.7)	15(7.1)	0.511
Antibiotic therapy, n(%)	169(70.7)	20(74.1)	149(70.0)	0.685
Remdesivir	135(56.5)	3(11.1)	132(62.3)	0.000
Dexamethasone	165(69.0)	1(3.7)	164(77.4)	0.000
Tocilizumab	66(27.6)	0(0)	66(31.1)	0.000
**Disease severity on admission**				
Mild	56(23.4)	6(22.2)	50(23.6)	0.233
Moderate	43(18.0)	10(37.0)	33(15.6)	
Severe	100(41.8)	8(29.6)	92(43.4)	
Critical	40(16.7)	3(11.1)	37(17.5)	

Data are expressed as n (%) and mean ± SD. **Abbreviations:** COVID-19: coronavirus disease 2019; BMI: body mass index; CAD: coronary arterial disease; CVA: cerebrovascular accident; DM: diabetes mellitus; CKD: chronic kidney disease; COPD: chronic obstructive pulmonary disease; HIV: human immunodeficiency virus.

**Table 2:** Baseline Laboratory data of patients with COVID-19

	Total n = 239	D614G Strain n = 27	Alpha Variant n = 212	p value
WBC, 1,000/μL	6.4 ± 5.3	6.6 ± 2.0	6.5 ± 5.6	0.864
Lymphocyte count	1120.7 ± 737.7	1613.4 ± 1049.5	1058.2 ± 666.1	0.014*
Platelet, 1,000/μL	216.2 ± 97.0	229.6 ± 100.5	214 ± 96.7	0.467
PT, seconds	14.1 ± 29.3	11.9 ± 0.5	14.3 ± 30.6	0.749
aPTT, seconds	29.5 ± 4.3	29.8 ± 3.8	29.4 ± 4.44	0.730
D-dimer, mg/L	1383.0 ± 2442.3	1236.8 ± 2265.8	1394.8 ± 2461.0	0.750
BUN, mg/dL	17.2 ± 13.1	16.4 ± 22.9	17.3 ± 11.4	0.715
Creatinine, mg/dL	1.04 ± 1.36	1.11 ± 2.04	1.03 ± 1.2	0.773
eGFR	86.2 ± 38.0	101.3 ± 49.5	84.3 ± 36.0	0.032
AST, U/L	39.4 ± 29.2	34.0 ± 20.0	40.0 ± 30.1	0.350
ALT, U/L	37.0 ± 37.1	30.2 ± 21.9	37.8 ± 38.3	0.365
Total bilirubin, mg/dL	0.49 ± 0.27	0.52 ± 0.3	0.49 ± 0.27	0.778
Na, mEq/L	136.3 ± 4.5	137.9 ± 4.5	136.1 ± 4.4	0.054
K, mEq/L	3.9 ± 1.9	3.6 ± 0.4	3.9 ± 2.0	0.526
LDH, U/L	362.0 ± 187.9	270.7 ± 142.5	371.1 ± 189.8	0.041*
CRP, mg/L	55.7 ± 69.1	42.5 ± 63.7	57.4 ± 69.7	0.348
Ferritin, ng/mL	916.5 ± 1396.0	751.0 ± 936.1	925.2 ± 1417.7	0.740
IL-6, pg/mL	72.48 ± 157.3	55.2 ± 101.4	74.6 ± 163.5	0.738

Data are expressed as mean ± SD. **Abbreviations:** WBC: white blood cell; PT: prothrombin time; aPTT: activated partial prothrombin time; BUN: blood urine nitrogen; AST: Aspartate Transaminase; ALT: alanine transaminase; LDH: lactate dehydrogenase; CRP: C‐reactive protein; IL-6: interleukine-6.

**Table 3:** Treatment and outcomes of COVID-19 patients

	Total n = 239	D614G Strain n = 27	Alpha Variant n = 212	Odds ratio 95% C.I.	p value
Disease progression, n(%)	94(39.3)	8(29.6)	86(40.6)	0.617(0.258-1.473)	0.261
Respiratory failure, n(%)	65(27.2)	5(18.5)	60(28.3)	0.576(0.208-1.590)	0.242
ARDS, n(%)	44(18.4)	3(11.1)	41(19.3)	0.521(0.150-1.815)	0.230
Admission to ICU, n(%)	64(26.8)	5(18.5)	59(278)	0.589(0.213-1.629)	0.187
Acute kidney injury, n(%)	47(19.7)	4(14.8)	43(20.3)	0.684(0.225-2.081)	0.503
Cardiac injury, n(%)	14(5.9)	1(3.7)	13(6.1)	0.589(0.74-4.687)	0.615
Duration from symptom to 1^st^ Ct No.>30, days	17.8 ± 12.1	14.0 ± 12.5	18.3 ± 12.0	-	0.112
Mortality, n(%)	17(7.1)	3(11.1)	14(6.6)	1.768(0.474-6.598)	0.393
Duration of hospitalization, days	23.0 ± 32.0	41.9 ± 81.1	20.7 ± 17.1	-	0.187
Duration of ICU stay, days	23.0 ± 22.0	31.6 ± 28.8	21.9 ± 21.0	-	0.357

Data are expressed as n (%) and mean ± SD. **Abbreviations:** ARDS: acute respiratory distress syndrome; ICU: intensive care unit; Ct: cycle threshold.

**Table 4:** Demographic characteristics and outcomes of patients with non-COVID-19 and COVID-19

	Non-COVID-19 n = 89	COVID-19 n = 239	p value
Age, year	62.7 ± 13.1	59.1 ±14.5	0.104
Male gender, n(%)	62 (69.7)	121(50.6)	0.001*
BMI	23.3 ± 5.4	25 ± 4.7	0.013*
Active smoker, n(%)	37 (41.6)	11(4.6)	<0.001*
**Co-morbidities**			
Hypertension, n(%)	35 (39.3)	89(37.2)	0.790
CAD, n(%)	3 (3.4)	35(14.6)	0.759
Heart failure, n(%)	0 (0)	10(4.2)	0.001*
Atrial fibrillation, n(%)	0 (0)	10(4.2)	0.001*
CVA, n(%)	24 (27.0)	12(5.0)	0.845
DM, n(%)	24 (27.0)	48(20.1)	0.223
CKD, n(%)	10 (11.2)	21(8.8)	0.524
COPD, n(%)	4 (4.5)	11(4.6)	0.949
Asthma, n(%)	24 (27.0)	7(2.9)	0.185
Immunosuppressive therapy, n(%)	32 (36.0)	3(1.3)	<0.001*
Malignancy, n(%)	43 (48.3)	9(3.8)	<0.001*
**Clinical outcomes**			
Respiratory failure, n(%)	65 (73.0)	65(27.2)	<0.001*
Admission to ICU, n(%)	65 (73.0)	64(26.8)	<0.001*
Length of ICU stay, days	26 ± 22	23 ± 22	0.553
Length of hospital stay, days	33.8 ± 31.1	23 ± 32	0.001*
Mortality, n(%)	49 (55.1)	17(7.1)	<0.001*

Data are expressed as n (%) and mean ± SD. **Abbreviations:** COVID-19: coronavirus disease 2019; BMI: body mass index; CAD: coronary arterial disease; CVA: cerebrovascular accident; DM: diabetes mellitus; CKD: chronic kidney disease; COPD: chronic obstructive pulmonary disease; ICU: intensive care unit.

**Table 5:** Risk factors associated with delayed viral RNA clearance

Variable	Univariate Analysis			Multivariate Analysis		
	HR	95% CI	p value	HR	95% CI	p value
D614G Strain (ref. Alpha strain)	0.27	0.11-0.68	0.005	0.20	0.49-1.21	0.097
Age>65 years	0.66	0.43-1.02	0.064	0.66	0.41-1.06	0.090
Obesity (BMI>30)	1.35	0.64-2.81	0.423			
Current/Ex-smoker	0.82	0.42-1.64	0.599			
Hypertension	0.69	0.45-1.07	0.105			
Coronary Artery Disease	0.95	0.58-1.55	0.838			
Heart failure	0.79	0.24-2.52	0.691			
Arrhythmia	0.51	0.15-1.68	0.273			
Cerebrovascular Accident	0.41	0.16-1.03	0.059	0.69	0.26-1.82	0.464
Chronic Kidney Disease	1.09	0.57-2.06	0.782			
Diabetes mellitus	0.99	0.59-1.65	0.988			
COPD	1.13	0.54-2.35	0.737			
Asthma	2.11	0.77-5.81	0.145			
Autoimmune Disease	4.16	1.30-13.3	0.004	3.98	1.21-13.04	0.022*
HIV	1.47	0.20-10.89	0.702			
Pneumonia at admission	0.74	0.45-1.21	0.234			
Dexamethasone therapy	1.69	0.98-2.91	0.057	2.24	0.98-5.11	0.054
Tociluzimab therapy	0.86	0.56-1.31	0.493			
Remdesivir therapy	1.37	0.88-2.13	0.160			
Antibiotics Therapy	0.38	0.22-5.81	0.001	0.26	0.13-0.55	<0.001*

**Abbreviations:** COPD: chronic obstructive pulmonary disease; HIV: human immunodeficiency virus; HR: hazard ratio.

**Table 6:** Risk factors associated with hospital mortality

Variable	Univariate Analysis			Multivariate Analysis		
	HR	95% CI	p value	HR	95% CI	p value
D614G Strain (ref. Alpha strain)	1.20	0.342-4.25	0.770			
Age>65	8.55	1.93-37.72	0.005	5.14	1.06-24.72	0.041*
Obesity (BMI>30)	2.12	0.59-7.57	0.244			
Current/ex-smoker	0.48	0.60-3.73	0.490			
HTN	1.48	0.56-3.90	0.421			
Coronary Artery Disease	2.79	1.04-7.46	0.041	1.76	0.49-6.24	0.378
Heart failure	3.20	0.72-14.22	0.125			
Arrhythmia	3.06	0.69-13.41	0.138			
Cerebrovascular accident	6.81	2.41-19.18	<0.001	3.62	1.25-10.42	0.017*
Diabetes mellitus	2.14	0.79-5.82	0.132			
CKD	3.65	1.26-10.56	0.016	1.31	0.32-5.30	0.697
COPD	1.75	0.39-7.78	0.456			
Pneumonia at admission	2.33	0.52-10.31	0.263			
Dexamethasone therapy	1.26	0.412-3.88	0.682			
Tociluzimab therapy	1.23	0.45-3.29	0.681			
Remdesivir therapy	1.22	0.45-3.30	0.695			

**Abbreviations:** COPD: chronic obstructive pulmonary disease; CXR: Chest X-ray; HR: hazard ratio.
